# Statistical shape modeling of the hip and the association with hip osteoarthritis: a systematic review

**DOI:** 10.1016/j.joca.2020.12.003

**Published:** 2020-12-15

**Authors:** M.M.A. van Buuren, N.K. Arden, S.M.A. Bierma-Zeinstra, W.M. Bramer, N.C. Casartelli, D.T. Felson, G. Jones, N.E. Lane, C. Lindner, N.A. Maffiuletti, J.B.J. van Meurs, A.E. Nelson, M.C. Nevitt, P.L. Valenzuela, J.A.N. Verhaar, H. Weinans, R. Agricola

**Affiliations:** † Department of Orthopedics, Erasmus MC, University Medical Center Rotterdam, Rotterdam, the Netherlands; ‡ Nuffield Department of Orthopedics, Rheumatology and Musculoskeletal Sciences, University of Oxford, Oxford, UK; § NIHR Musculoskeletal Biomedical Research Unit, Arthritis Research UK Centre for Sport, Exercise, and Osteoarthritis, University of Oxford, Oxford, UK; ║ Department of General Practice and Department of Orthopedics, Erasmus MC, University Medical Center Rotterdam, Rotterdam, the Netherlands; ¶ Medical Library, Erasmus MC, University Medical Center Rotterdam, Rotterdam, the Netherlands; # Human Performance Lab, Schulthess Clinic, Zürich, Switzerland; †† Laboratory of Exercise and Health, ETH Zürich, Schwerzenbach, Switzerland; ‡‡ Centre for Epidemiology Versus Arthritis, Faculty of Biology, Medicine and Health, Manchester Academic Health Science Centre, The University of Manchester, Manchester, UK; §§ NIHR Manchester Biomedical Research Centre, Manchester University NHS Foundation Trust, Manchester Academic Health Science Centre, Manchester, UK; ║║ Department of Rheumatology, Boston University School of Medicine, Boston, MA, USA; ¶¶ Menzies Institute for Medical Research, University of Tasmania, Hobart, Tasmania, Australia; ## Department of Medicine, University of California, Davis, CA, USA; ††† Division of Informatics, Imaging & Data Sciences, University of Manchester, Manchester, UK; ‡‡‡ Department of Internal Medicine, Erasmus MC, University Medical Center Rotterdam, Rotterdam, the Netherlands; §§§ Thurston Arthritis Research Center and Department of Medicine, University of North Carolina, Chapel Hill, NC, USA; ║║║ Department of Epidemiology and Biostatistics, University of California, San Francisco, CA, USA; ¶¶¶ Department of Systems Biology, University of Alcala´, Madrid, Spain; ### Department of Orthopedics, University Medical Center Utrecht, Utrecht, the Netherlands; †††† Department of Biomechanical Engineering, Delft University of Technology, Delft, the Netherlands

**Keywords:** Coxa valga, Coxa vara, Femoroacetabular impingement, Pincer, Anatomy, Epidemiology

## Abstract

**Objective:**

To summarize available evidence on the association between hip shape as quantified by statistical shape modeling (SSM) and the incidence or progression of hip osteoarthritis.

**Design:**

We conducted a systematic search of five electronic databases, based on a registered protocol (available: PROSPERO CRD42020145411). Articles presenting original data on the longitudinal relationship between radiographic hip shape (quantified by SSM) and hip OA were eligible. Quantitative meta-analysis was precluded because of the use of different SSM models across studies. We used the Newcastle–Ottawa Scale (NOS) for risk of bias assessment.

**Results:**

Nine studies (6,483 hips analyzed with SSM) were included in this review. The SSM models used to describe hip shape ranged from 16 points on the femoral head to 85 points on the proximal femur and hemipelvis. Multiple hip shape features and combinations thereof were associated with incident or progressive hip OA. Shape variants that seemed to be consistently associated with hip OA across studies were acetabular dysplasia, cam morphology, and deviations in acetabular version (either excessive anteversion or retroversion).

**Conclusions:**

Various radiographic, SSM-defined hip shape features are associated with hip OA. Some hip shape features only seem to increase the risk for hip OA when combined together. The heterogeneity of the used SSM models across studies precludes the estimation of pooled effect sizes. Further studies using the same SSM model and definition of hip OA are needed to allow for the comparison of outcomes across studies, and to validate the found associations.

## Introduction

Hip osteoarthritis (OA) is one of the most common types of OA, and is a major contributor to the number of years lived with disability worldwide^1^. Hip shape has been recognized as an important risk factor for hip OA^2^. For this reason, the influence of hip shape has been increasingly studied over the last decade^3–9^. Hip shape variants that are known to significantly increase the risk for hip OA are acetabular dysplasia and cam morphology^2,7,10^. These hip shape variations are typically quantified by predefined radiological measurements such as the center-edge angle (CEA) and the alpha angle. However, other hip shape variants that are currently not captured by predefined radiological measurements may also play a role in the etiology of hip OA. The sole use of predefined measurements for hip shape analysis may therefore impede the discovery of further hip shape variants that increase the risk for hip OA.

This limitation has been partially circumvented by the emergence of statistical shape modeling (SSM)^11^ as a novel shape analysis technique. SSM allows quantification of the whole shape of the hip and/or pelvis, in contrast to predefined measurements^12,13^. The application of SSM yields a set of shape variants, called shape modes, that are present in the studied population. When SSM is applied to radiographic images of the hip, the association between each hip shape mode and hip OA can be measured.

SSM has been increasingly used, and many different hip shape modes have so far been associated with hip OA. However, the interpretation of the SSM shape modes can be difficult and there is no thorough overview of the related literature yet. The purpose of this systematic review was to summarize which hip shape variants were found to be associated with incident or progressive hip OA, and to determine if there are any consistent patterns of similar shape variants to be recognized across different studies.

## Methods

### Protocol and registration

We reported this systematic review according to the Preferred Reporting Items for Systematic Reviews and Meta-Analyses (PRISMA) guidelines^14^. The review protocol was first submitted to PROSPERO on September 23, 2019, and was registered on April 28, 2020 (available from: www.crd.york.ac.uk/prospero/display_record.php?ID=CRD42020145411).

### Eligibility criteria

All publications presenting original research on the association between hip shape and hip OA in human adults were considered eligible, as were conference abstracts published in 2016 or later. The inclusion criteria were:

- Assessment of the longitudinal association between hip shape and OA had to be an aim of the study;
- Hip shape had to be assessed with some form of SSM;
- Hip OA should be either incident or progressive;
- The definition of hip OA could be radiological, clinical, by total hip replacement (THR) status, or a combination of those;
- Studies had to have control subjects that did not develop incident or progressive hip OA during the study.

The exclusion criteria were:

- Hip shape was measured contralaterally to the hip that developed the outcome (e.g., the shape of the contralateral hip in case of THR);
- The studied hip shape variant was explicitly described to be secondary to other conditions (e.g., childhood hip disease, trauma, avascular necrosis, tumors, previous hip surgery);
- The primary outcome was biomechanical injury, or the validation of a novel diagnostic technique;
- The OA outcome reflected ‘early osteoarthritic changes’, such as cartilage damage during arthroscopy or novel magnetic resonance imaging (MRI) techniques like delayed gadolinium-enhanced MRI of cartilage (dGEMRIC), Scoring Hip Osteoarthritis with MRI (SHOMRI), and ρ mapping.

### Search and deduplication

An experienced information specialist (WB) searched the databases Embase (via Embase.com, since 1971), MEDLINE (Medline ALL via Ovid, since 1946), Web of Science Core Collection (since 1975) and the Cochrane Central Register of Trials (via Wiley, since 1992) from inception until April 25, 2020 (date last searched). A previously published method was used for search development and optimization^15^. The searches combine terms (both thesaurus terms where available, and terms in title and/or abstract) for hip osteoarthritis with terms for anatomy or morphology and terms for risk or pathology. Search results were limited to exclude (1) animal and child-only studies, (2) conference abstracts published before 2016, and (3) publications in other languages than English. The full search strategy can be found in Supplement 1. Additionally, we searched Google Scholar and screened the reference lists of the included references for any other relevant articles. The search results from all databases were imported in EndNote and deduplicated^16^.

### Study selection

Two reviewers (MvB and RA) independently screened the titles and abstracts of all search results, and after having compared the included references, independently reviewed the full text of all potentially eligible studies. This process was done in EndNote with a predefined method^17^. Subsequently the reviewers held a consensus meeting to discuss each full-text article separately, and to select the final studies to be included. A third reviewer (MN) was consulted to resolve any disagreements.

### Data collection/extraction

A custom open-ended electronic data extraction form was developed and pilot-tested with a sample of the included studies. The used data extraction form, including the full list of extracted variables, can be found in Supplement 2. Data extraction was independently performed in duplicate by two reviewers (MvB and RA), and the results were compared in a consensus meeting. For one conference abstract of which the full text was not published yet, the reviewers requested and received the full text manuscript from the authors.

### Risk of bias assessment

We used the Newcastle–Ottawa Scale (NOS) to assess the risk of bias of the individual studies^18^. We used either the cohort version or the case–control version as appropriate. The questions and the scoring key can be found in Supplement 3. The two reviewers (MvB and RA) independently appraised the quality of the individual studies, and disagreements were resolved in a consensus meeting. Publication bias was reduced by searching for recent conference abstracts and by searching Google Scholar for gray literature.

### Statistical shape analysis

The application of SSM requires all images (e.g., radiographs) to be annotated by placing a set of points around the outline of the bone. To negate the effect of size and orientation, the outline of the bone (the shape) across images is usually aligned first using a technique called Procrustes analysis. Principal component analysis (PCA) is then applied to identify the main variations in shape (called shape modes) within the given population (i.e., across all images), summarized as a statistical shape model. Shape modes are stored as a set of continuous variables, usually standardized to have a mean of 0 and a standard deviation of 1, and are linearly independent of each other. These shape modes represent the apparent radiographic shape, and may not always match the true anatomic shape due to the influence of subject positioning and radiographic projection effects. Shape modes are ordered by their contribution to the total shape variance, the lower mode numbers being the most contributing. Because the SSM process arbitrarily assigns deviations from the mean shape as either positive or negative, a certain shape variant can either be positively or negatively (inversely) associated with the outcome. Furthermore, due to the nature of PCA the definition of individual shape modes will be data dependent and thus will vary across datasets/studies.

### Data synthesis

The main outcome measures that we extracted were the measures of association for the relationship between SSM-defined hip shape and OA. These could be odds ratios (OR), relative risk (RR), prevalence ratios (PR), or any other association measures. If present, the covariate-adjusted measures were extracted. We only performed qualitative data synthesis, as the use of SSM models resulting from different studies precludes statistical pooling and thus meta-analysis. To still be able to summarize associations, we qualitatively compared the descriptions (as provided in the original papers) of the different hip shape modes from across studies. The reported shape descriptions are therefore either the literal descriptions by the original authors, or the reviewers’ interpretation of the original figures if these were unambiguous. If neither was the case, we did not report a shape description.

## Results

### Study selection

**Fig. 1:**
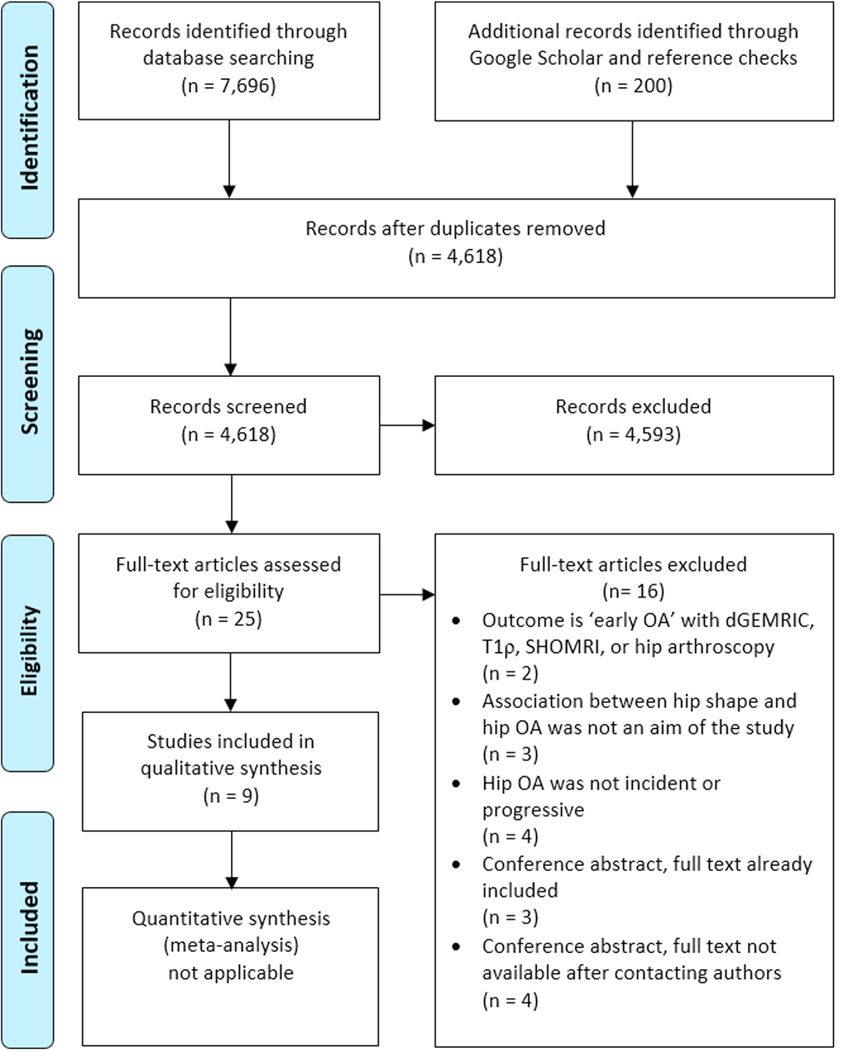
PRISMA flow diagram detailing the literature search, screening and inclusion process. PRISMA, Preferred Reporting Items for Systematic Reviews and Meta-Analyses; OA, osteoarthritis; dGEMRIC, delayed gadolinium-enhanced magnetic resonance imaging of cartilage; SHOMRI, scoring hip osteoarthritis with magnetic resonance imaging.

The initial database searches yielded 4,618 unique references, which were screened by title and abstract. Twenty-five of these had used SSM to quantify hip shape and were retrieved for full-text reading. The screening and inclusion process as well as the reasons for exclusion are shown in Fig. 1. Finally, we included nine articles in this review^19–27^.

### Study characteristics

The main characteristics of the nine included studies (published between 2007 and 2017) are presented in Table I. The study by Mezhov *et al.*^27^ has only been published as a conference abstract as of yet, but we received the full manuscript from the authors upon request. The included studies present data on a total population of 4,706 subjects, with 6,483 hips analyzed with SSM. Not all subjects were unique, since some parts of study populations were used in two separate articles^20,23,25,27^. The Rotterdam Study population was also used twice, but random samples were drawn, making duplicate entry of subjects unlikely^19,24^. Factoring in the use of data from these study populations in separate articles, the number of unique hips analyzed with SSM was 4,584. Median sample size was 664 subjects (range 110–831) and median follow-up period was 6.5 years (range 5–19). The overall proportion of females was 69.0%, ranging from 51%^23,27^ to 100%^20,26^. The mean age of included subjects ranged from 53.6^20^ to 70.7^26^, with a pooled mean age of 61.8 years across all studies.

### Risk of bias

A summary of the risk of bias assessment is presented in Table II, whereas an extensive overview can be found in Supplement 2. Eight of the included studies were deemed as having good methodological quality, with a low risk of bias^19–22,24–27^. When strictly following the NOS guidelines, one study scored poorly because of self-reported THR assessment and the lost to follow-up rate^23^. However, the reviewers considered the overall quality of this study sufficient to regard the findings as reliable.

### Assessment of exposure and outcome

An overview of the assessment of exposure and outcome in each study can be found in Table III. Seven studies^19–22,24–26^ used pelvic radiographs to assess hip shape, whereas the other two^23,27^ used Dual-energy X-ray absorptiometry (DXA). The SSM points used to outline the hip shape varied from 16^19^ to 85^23,27^. Three studies only described the femoral head^19^ or part of the proximal femur^21,26^. Three studies additionally included the acetabular roof^22,23,27^. The remaining three studies also included the ipsilateral lower pelvis, consisting of the acetabulum, the pelvic teardrop, and the pubic and ischial bones^20,24,25^. All studies^19–27^ used the ASM toolkit (University of Manchester, Manchester, UK) to annotate the images. Seven studies also used this toolkit to create the SSM, while two studies^23,27^ additionally used SHAPE software (University of Aberdeen, Aberdeen, UK) for this. Both the ASM toolkit and the SHAPE software are based on Procrustes analysis and PCA.

Eight studies^19,20,22–27^ used THR as a definition for hip OA. Other used definitions were Kellgren–Lawrence (KL) grade ≥2^21,24^, an increase in KL grade of ≥3 points compared to baseline^19^, Croft grade ≥2^26^, and meeting the American College of Rheumatology (ACR) criteria^25^. Some studies included multiple definitions of hip OA, either creating subgroups per outcome definition^19,25^ or pooling multiple definitions into one group^24,26^. Six studies^19–21,24–26^ used incident hip OA as the outcome, meaning all cases had baseline OA scores (e.g., KL, Croft) of 0e1. In the remaining three studies^22,23,27^, the distinction between incidence and progression could not be made because part of the study sample already had OA scores ≥2 at baseline. All studies corrected for two or more covariates in their analyses^19–26^.

### The association between hip shape and THR

The results from the studies that used THR as a separate outcome definition^19,20,22,23,25,27^ are summarized in Table IV, whereas the complete results (including non-significant associations) can be found in Supplement 2. All six studies that used THR as a separate outcome measure found at least one shape mode that was statistically significantly associated with THR (median 2 modes, range 1–6) at the chosen alpha level. The indication for THR was incident hip OA in three studies^19,20,25^, and incident or progressive hip OA in the other three studies^22,23,27^. One study^25^ used Bonferroni correction for multiple testing.

A total of 18 hip shape modes were associated with future THR across the different studies. One of these modes (describing a flattened head–neck junction, a flat major trochanter and a prominent acetabular posterior wall) showed a consistent association in two different populations, namely the CHECK and Chingford populations^20^. Five studies^19,20,23,25,27^ (out of the six that used THR as a separate outcome measure) found at least one shape mode consistent with cam morphology; and four^20,22,23,27^ out of six studies found a mode representing acetabular dysplasia. A hip shape variant possibly representing pincer morphology was associated with THR in one study^25^ out of the six studies that included the acetabular roof in their model^20,22–25,27^. The description of this shape mode was “more pronounced lateral acetabular rim” in this study. Deviations in acetabular version were associated with THR in both studies that included the acetabulum in their shape model^20,25^. One study describes a shape mode with “a prominent acetabular posterior wall”, possibly representing excessive acetabular anteversion, combined with “a flattened head–neck junction and a flat major trochanter”^20^. The other study describes a mode with “acetabular retroversion”, combined with a “flat head–neck junction and broad femoral neck”^25^.

### The association between hip shape and radiographic hip OA

Studies that mainly used radiographic hip OA as outcome measure^19,21,24,26^ are summarized in Table V, whereas the complete results (including non-significant associations) can be found in Supplement 2. At least one shape mode per study was statistically significantly associated with hip OA (median 3 modes, range 1–6) at the chosen alpha level. In all four studies the outcome was incident hip OA (baseline OA scores of 0–1). Two studies^24,26^ used a combined definition of hip OA, where THR and radiographic hip OA were pooled into a single endpoint. However, one of those studies only seemed to present radiographic hip OA cases in their results, and no THRs^24^. Two studies^24,26^ used Bonferroni correction for multiple testing.

Thirteen hip shape modes were associated with incident radiographic hip OA. One study^21^ presented two hip shape modes that showed different associations in different subgroups. In this study, mode 2 (representing alterations in the transition between greater trochanter and femoral neck and femoral neck length and thickness) was inversely associated with symptomatic radiographic hip OA in the entire study population, but positively associated with radiographic hip OA in males only. Positive mode 2 scores represented flattening of the femoral head, suggestive of cam morphology. Two^19,21^ out of four studies found shape modes representing cam morphology; and the only study that included the acetabulum in their model^24^ found shape modes representing dysplasia. Acetabular version was also associated with radiographic hip OA in that study, but the type (ante- or retroversion) was unspecified^24^.

### The association between hip shape and clinical hip OA

One study^25^ used a clinical definition of hip OA, namely the ACR criteria, next to another definition (THR). They found no statistically significant associations between baseline hip shape modes and ACR criteria at follow-up. Another study^21^ made the distinction between symptomatic radiographic hip OA and overall radiographic hip OA. This study found associations between different shape modes and symptomatic radiographic hip OA in the overall population, as well as in subgroups with or without baseline symptoms (Table V).

## Discussion

In this systematic review we have summarized all available evidence from the published literature on the association between SSM-defined apparent radiographic hip shape and hip OA. Our results show that every published study on this topic that was included in this review found at least one hip shape mode statistically significantly associated with incident or progressive hip OA or future THR. Most studies found multiple (up to six) linearly independent hip shape modes associated with hip OA. Most of the included studies used different populations and different SSM point positions for their modeling, which complicates the comparison of hip shape modes between studies. However, in the following we attempt to discuss the overall patterns in radiographic hip shape that were found to be associated with hip OA.

Shape variants that likely represent cam morphology and acetabular dysplasia were consistently found to be associated with future THR and/or incidence or progression of radiographic hip OA. Shape modes that might represent cam morphology were described as “cam-type change of femoroacetabular impingement”^21^, “pistol-grip deformity”^23^, “less concavity of superior head–neck junction”^25^, “less pronounced curve from upper femoral neck into the head”^19^, “less head-neck offset”^25^, “non-spherical femoral head”^20,23,27^, “flattening of the head-neck transition”^21^, and “flattening of the femoral head”^21^. Modes that may represent acetabular dysplasia were described as “less/poor/decreasing acetabular coverage”^22–24,27^, and “a shallow acetabulum”^20^. The associations between hip OA and both cam morphology and acetabular dysplasia have already been proven in other studies that used traditional measurements, such as the alpha angle and the CEA^7,8,10,28–35^. Two cross-sectional studies that used SSM also found associations between cam morphology and the presence of hip OA^36,37^. These studies were not included in our systematic review due to their cross-sectional design. Because there were no baseline OA measurements, it remains unclear whether the shape modes found in these studies preceded hip OA or resulted from it.

A shape mode possibly representing pincer morphology was also associated with THR in one of the studies included in this review^25^. Other studies, using traditional measurements such as the CEA and the crossover sign, did not find a positive association between pincer morphology and hip OA so far^7,8,10^. Maybe the risk for hip OA is only increased when a pincer morphology is mixed with other shape features, or for certain subtypes of pincer morphology that are not captured with traditional measurements. A cross-sectional study^36^ (excluded from our systematic review) also found an association between pincer morphology and hip OA. In the shape mode of that particular study, the “pincer-type variation” was combined with a “larger femoral head and wider femoral neck”. This combination could theoretically aggravate femoroacetabular impingement. However, since no baseline OA measurements were done in this study, the “pincer-type variation” shape mode could have also represented an osteophyte of the lateral acetabulum, secondary to hip OA.

Multiple studies included in this systematic review found associations between acetabular version and hip OA. This is in line with studies using traditional measurements, which have also suggested that both acetabular anteversion and retroversion could be associated with hip OA^38–40^. A cross-sectional study that used SSM to define hip shape also found associations between two shape modes, possibly representing acetabular retroversion and anteversion respectively, and the presence of hip OA^41^.

Because one statistical shape mode often consists of more than one shape feature, extra caution has to be taken when singling out just one shape feature. The association with hip OA may only be present when there is a combination of multiple shape features. This is precisely the advantage of SSM. One combination that consistently appears to be associated with hip OA is cam morphology combined with dysplastic acetabular features^20,25^, a combination that has been previously described in the literature^42,43^. It is still not entirely clear why this combination would increase the risk for hip OA, because theoretically a cam would be less likely to impinge with a dysplastic acetabulum. However, one computer simulation study has demonstrated that impingement can still occur, but more proximally and more medially than with a normal acetabulum^44^. It remains unknown whether the higher risk is due to the cam morphology alone, the dysplastic acetabulum, or the interaction between the two. Another reported shape combination was the presence of a cam morphology with acetabular retroversion^25^, which could be theoretically explained by femoroacetabular impingement happening earlier during hip flexion and internal rotation. The combination of a valgus hip with acetabular dysplasia^22,23^ was associated with hip OA in two studies. From a biomechanical perspective, this could be explained by higher vertical joint reaction force^45^ acting on a smaller surface during weight bearing. This combination has also been previously described^43^. Besides the aforementioned combinations, variations in the size of the trochanters, the length and width of the femoral neck, and the apparent rotation of the femur and pelvis were found, but no obvious patterns were seen in these variations.

The magnitude of the reported associations between hip shape modes and hip OA varied greatly between studies. Due to the different SSM point positions and different outcome definitions, the association measures are not directly comparable. Large ORs or RRs can be interpreted as a strong association nevertheless.

### Strengths and limitations

This is the first systematic review on the association between SSM-defined radiographic hip shape and hip OA. It offers an overview of the patterns of hip shape features that are associated with hip OA in multiple populations. The interpretation and implications of the results were carefully discussed within the review group, which contains experts in the fields of both hip OA and SSM. Strengths of the included studies are the relatively large sample sizes and the various populations of differing ages and ethnicities that were included. Overall, the included studies scored well on methodological quality.

One limitation of this review is that we were not able to conduct a meta-analysis. This is inherent to SSM, because the shape modes will be defined by the population from which they were created. This was already taken into account when designing the review protocol. The lack of a meta-analysis makes validation of associations difficult. We therefore subjectively described patterns of hip shape that seemed to be consistently associated with hip OA across the included studies. A second limitation is that the interpretation and description of the shape modes are relatively subjective processes, which were left to the authors of the included papers. Still, we purposefully reported only the literal descriptions from the original articles to reduce bias by our own interpretation. Another limitation is that none of the included studies have validated the found associations in an independent test dataset. Internal validation would have been possible if the datasets had been divided into a training set and a test set. This is something that future SSM studies could possibly address. One more consideration is the influence that hip OA may have on hip shape. As some studies have shown, hip OA may not only result from certain hip shape variants but can also cause changes in hip shape^46^. This is not a problem in incidence studies^19–21,24–26^ where all analyzed hips were free of OA at baseline, but the hip shape modes found in progression studies^22,23,27^ could already be a result of early hip OA. Further limitations of the included studies are the heterogeneity of pelvic radiograph protocols and outcome definitions, and the varying use of covariate adjustment. Further research is required to investigate whether significant covariates (e.g., gender) may require independent shape models instead of simply adjusting for them. Lastly, most studies only described shape modes that were significantly associated with hip OA at their chosen alpha level, but some studies used Bonferroni correction, whereas others did not. This may have led to some reporting bias, even more so because statistical significance does not always translate to clinical significance. In our opinion, the use of multiple testing correction in SSM analysis should depend on the goal of the analysis. When SSM is used for hypothesis generation, you could argue not using a correction because you would want to find any possible leads. The associations found in this way should not be taken as evidence though, but have to be investigated further. In other cases, a method like the Bonferroni correction is warranted. In any case, authors should preferably explain their reasoning for (not) using multiple testing correction.

## Conclusion

This systematic review suggests that several radiographic hip shape features and combinations thereof are associated with the incidence or progression of radiographic hip OA and with future THR. Associations of both cam morphology and acetabular dysplasia with hip OA have been found by SSM in multiple studies. In addition, hip shape features other than these well-known variants also appear to be associated with hip OA. Moreover, certain combinations of (sometimes subtle) hip shape features, rather than single features, may increase the risk for development or progression of hip OA when present together. More research with SSM is needed to validate these associations, and a standardized set of SSM point positions should be used to allow comparison between studies. When SSM is used to generate hypotheses, the found associations could be tested with traditional radiographic measurements in an independent sample. This would both validate the associations and make them more easily transferrable to clinical practice.

## Supplementary Material

1

2

3

Supplementary data to this article can be found online at https://doi.org/10.1016/j.joca.2020.12.003.

## Figures and Tables

**Table I:** Characteristics of the nine included studies

Study	Country	Study population	Study design	N subjects	N hips	Age in years, mean (SD)	% Females	Mean follow-up

Agricola *et al.* (2015)^20^	Netherlands	CHECK study	Prospective cohort	550	1,100	55.8 (5.1)	100%	5 years
	UK	Chingford study	Nested case-control	114	114	53.6 (5.4)	100%	19 years
Agricola *et al.* (2013)^25^	Netherlands	CHECK study	Prospective cohort	723	1,411	55.9 (5.2)	79%	5 years
Ahedi *et al.* (2017)^23^	Australia	TASOAC study	Prospective cohort	831	831	63.2 (7.5)	51%	10 years
Barr *etal.* (2012)^22^	UK	PCR study	Nested case-control	195	102	62.7 (10.7)	68%	5 years
Castaño-Betancourt *et al.* (2013)^24^	Netherlands	Rotterdam Study	Prospective cohort	688	1,283	65.6*	58%	6.5 years
Gregory *et al.* (2007)^19^	Netherlands	Rotterdam Study	Nested case-control	110	110	68.7 (5.9)†	75%	6 years
Lynch *et al.* (2009)^26^	USA	SOF	Nested case-control	351	351	70.7 (4.4)†	100%	8.3 years
Mezhov *et al.*^27^	Australia	TASOAC study	Prospective cohort	802	799	62.5 (7.3)†	51%	12.1 years
Nelson *et al.* (2014)^21^	USA	JoCoOA project	Nested case-control	342	382	61.7 (9.0)	61%	6 years

UK, United Kingdom; USA, United States of America; CHECK, Cohort Hip and Cohort Knee; TASOAC, Tasmanian Older Adults Cohort; PCR, Primary Care Rheumatology; SOF, Study of Osteoporotic Fractures; JoCoOA, Johnston County Osteoarthritis; SD, standard deviation.

* No measure of variability was reported.

† Pooled mean and SD calculated by reviewers.

**Table II:** Newcastle—Ottawa Scale for risk of bias assessment

Study	NOS version	Selection	Comparability	Exposure/Outcome	Total stars	Quality Score†

Agricola *et al.* (2015)^20^	Case-Control*	★★★★	★★	★★★	9	Good
	Cohort*	★★★★	★★	★★★	9	Good
Agricola *et al.* (2013)^25^	Cohort	★★★★	★★	★★★	9	Good
Ahedi *et al.* (2017)^23^	Cohort	★★★★	★★	☆★☆	7	Poor
Barr *et al.* (2012)^22^	Case-Control	★☆★★	★★	★★☆	7	Good
Castano-Betancourt *et al.* (2013)^24^	Cohort	★★★★	★★	★★★	9	Good
Gregory *et al.* (2007)^19^	Case-Control	★★★★	★☆	★★★	8	Good
Lynch *et al.* (2009)^26^	Case-Control	★☆★★	★★	★★★	8	Good
Mezhov *et* al.^27^	Cohort	★★★★	★☆	★★☆	7	Good
Nelson *et al.* (2014)^21^	Case-Control	★★★★	★★	★★★	9	Good

See Supplement 2 for the reviewers’ considerations for each question. See Supplement 3 for score calculation. NOS, Newcastle–Ottawa Scale.

* Two versions of NOS were used: NOS case-control for the Chingford population, and NOS cohort for the Cohort Hip and Cohort Knee population.

† The table shows the stars earned for each domain, and the total amount of stars.

**Table III:** Overview of the exposure and outcome assessments used in the included studies

Study	Exposure assessment				Outcome assessment		
	Imaging modality	N points in SSM model	Anatomical regions included in SSM model	Software used for SSM	Protocol pelvic radiograph	Hip OA definition	Hip OA type

Agricola *et al.* (2015)^20^	X-ray	75^a^	Proximal femur and lower pelvis	ASM toolkit	AP weight-bearing, 15° IR^1^ AP supine, neutral^2^	THR	Incident
Agricola *et al.* (2013)^25^	X-ray	75^a^	Proximal femur and lower pelvis	ASM toolkit	AP weight-bearing, 15° IR	THR/ACR criteria*	Incident
Ahedi *et al.* (2017)^23^	DXA	85^b^	Proximal femur and acetabular roof	ASM toolkit + SHAPE	AP weight-bearing, 10° IR	THR	Incident & progressive
Barr *et al.* (2012)^22^	X-ray	16^c^ 45	Femoral head and superior neck Proximal femur and acetabular roof	ASM toolkit	AP unspecified	THR	Incident & progressive
Castaño-Betancourt *et al.* (2013)^24^	X-ray	67	Proximal femur and lower pelvis	ASM toolkit	AP weight-bearing, 10° IR	THR/KL ≥ 2**	Incident
Gregory *et al.* (2007)^19^	X-ray	16^c^	Femoral head and superior neck	ASM toolkit	AP weight-bearing, 10° IR	THR/KL increase of ≥3 points*	Incident
Lynch *et al.* (2009)^26^	X-ray	60^d^	Proximal femur	ASM toolkit	AP supine, 15 −30° IR	THR/Croft ≥2**	Incident
Mezhov *et al.*^27^	DXA	85^b^	Proximal femur and acetabular roof	ASM toolkit + SHAPE	AP weight-bearing, 10° IR	THR	Incident & progressive
Nelson *et al.* (2014)^21^	X-ray	60^d^	Proximal femur	ASM toolkit	AP supine, 15° IR	KL ≥ 2	Incident

a,b,c,d These pairs of studies used the same point set for annotation;

1 Protocol used in Cohort Hip and Cohort Knee (CHECK);

2 Protocol used in Chingford cohort;

* These studies used two definitions for hip OA and performed subgroup analyses for the separate outcomes;

** These studies used two definitions for hip OA and pooled these into one group; SSM, statistical shape modeling; OA, osteoarthritis; DXA, Dual-energy X-ray Absorptiometry; ASM, Active Shape Modelling; AP, anteroposterior; IR, internal rotation; THR, total hip replacement; KL, Kellgren–Lawrence grade, ACR, American College of Rheumatology criteria for hip OA.

**Table IV:** Hip shape modes significantly associated with total hip replacement outcome

Study	Association measure	Subgroup	Shape mode	Explained variance	Shape that is associated with total hip replacement*	Effect size (95% CI)	*P*-value	Alpha level	Covariates

Agricola *et al.* (2015)^20^	OR	Chingford	2	–	*Longer and narrower femoral neck*	1.61 (1.02–2.54)	0.042	0.05	–
			17	–	Flattened head–neck junction, a flat major trochanter and a prominent acetabular posterior wall	0.41 (0.23–0.82)	0.01		
		CHECK	4	–	Non-spherical femoral head, together with a shallow acetabulum	0.38 (0.20–0.69)	0.002	0.05	Age BMI Baseline KL
			11	–	*Smaller femoral head, smaller major trochanter*	2.18 (1.23–3.86)	0.008		
			15	–	Orientation of pelvis & greater trochanter *More medial projection of greater trochanter*	1.66 (1.02–2.68)	0.04		
			17	–	Flattened head–neck junction, a flat major trochanter, and a prominent acetabular posterior wall	0.51 (0.33–0.80)	0.003		
			22	–	*Less concavity superior head-neck junction*	1.90 (1.29–2.78)	0.001		
Agricola *et al.* (2013)^25^	OR	Overall	7	–	Shorter femoral neck	0.54 (0.38–0.78)	0.001	0.002	Age
			11	–	Flat head–neck junction, broad femoral neck, acetabular retroversion	1.78 (1.28–2.47)	0.001		BMI Gender
			12	–	*Less superior joint space width, more pronounced lateral acetabular rim*	2.10 (1.46–3.04)	<0.001		
			15	–	Wider femoral neck, less head-neck offset	1.90 (1.39–2.59)	<0.001		
			22	–	*Not described, not shown in figures*	0.59 (0.42–0.81)	0.001		
Ahedi *et al.* (2017)^23^	PR	Overall	2	14.0%	Greater neck-shaft angle, narrower femoral neck, smaller & flatter femoral head, less acetabular coverage	1.60(1.20–2.15)	<0.05**	0.05	Age BMI Gender
			4	6.0%	Wider femoral neck, larger femoral head, larger joint space width, loss of sphericity at transition superior neck to head (pistol-grip deformity)	0.63 (0.50–0.84)	<0.05**		
Barr *et al.* (2012)^22^	OR	45-point model	2	–	Poor acetabular coverage, steeper neck-shaft angle	0.17 (0.04–0.71)	<0.05**	0.05	Baseline KL Clinical factors† Geometrical factors‡
Gregory *et al.* (2007)^19^	OR	Overall	3	–	Sharp transition from femoral head to the upper neck	3.71 (1.33–10.4)§	0.012	0.05	Age Gender
			6	–	Less pronounced curve from upper femoral neck into the head, sharper transition from femoral head to the lower neck	2.35 (1.15–4.82)§	0.019		
Mezhov *et al.*^27^	RR	Overall	2	–	Decreasing acetabular coverage	1.57 (1.01–2.46)	<0.05**	0.05	WOMAC pain
			4	–	Non-spherical femoral head	0.65 (0.44–0.97)	<0.05**		OARSI grade

* These shapes are positively associated with the outcome, unless stated otherwise. For a visual impression of what these shape modes look like, we refer to the original articles. Effect sizes are shown per 1 SD increase in shape mode value. An effect size ratio between 0 and 1 indicates that the negative SDs are associated with the outcome, and ratios above 1 indicate that positive SDs are associated with the outcome. Descriptions in regular typeface are taken literally from the original papers, while descriptions in italics are interpreted from the figures of the original papers;

** Exact *P*-values were not given, but were under the alpha level of 0.05;

† Clinical factors: use of a stick, physical function (from WOMAC), duration of pain;

‡ Geometrical factors: acetabular depth, center-edge angle, baseline minimum joint space width and femoral head migration;

§ ORs are for OA *with* THR vs OA *without* THR; CI: confidence interval; OR: odds ratio; PR: prevalence ratio; RR: relative risk; CHECK: Cohort Hip and Cohort Knee; BMI: body mass index; KL: Kellgren-Lawrence grade; WOMAC: Western Ontario and McMaster Universities Osteoarthritis Index; OARSI: Osteoarthritis Research Society International; SD: standard deviation.

**Table V:** Hip shape modes significantly associated with radiographic hip osteoarthritis outcomes

Study	Association measure	Subgroup	Shape mode	Explained variance	Shape that is associated with radiographic hip osteoarthritis*	Effect size (95% CI)	*P*-value	Alpha level	Covariates

Castaño-Betancourt *et al.* (2013)^24^	OR	Overall^a^	5	–	Less covering of the femoral head by the acetabulum	0.65 (0.54–0.77)	<0.0001	0.0021	Age Gender
			9	–	Shorter femoral neck	1.40 (1.14–1.72)	0.001		BMI
		Baseline KL 0^a^	12	–	Variation in acetabular version with corresponding rotation of the femur†	1.69 (1.24–2.30)	0.00094		
Gregory *et al.* (2007)^19^	OR	Overall	6	–	Less pronounced curve from upper femoral neck into the head, sharper transition from femoral head to the lower neck	1.62 (1.08–2.45)	0.02	0.05	Age Gender
Lynch *et al.* (2009)^26^	OR	Overall^b^	3	8.9%	Larger femoral head, longer and thinner femoral neck relative to the size of the trochanters and shaft	1.73 (1.25–2.39)	<0.001	0.005	Age Height Hip BMD
			5	3.3%	Larger than average greater trochanter size, smaller femoral neck size relative to the average size of the femoral head and shaft	2.31 (1.63–3.28)	<0.001		
			9	0.8%	Large femoral head compared to femoral neck, more pronounced greater trochanter	1.81 (1.32–2.49)	<0.001		
Nelson *et al.* (2014)^21^	OR	Overall	2	16.0%	Alterations in the transition between greater trochanter and femoral neck, a slight reduction in femoral neck width, and a qualitative impression of a longer femoral neck compared to the mean shape	1.47 (1.03–2.08)§	<0.05**	0.05	Age Gender BMI Race Baseline KL
			3	12.5%	Alterations in the transition between greater trochanter and femoral neck, a somewhat flatter femoral head	1.54 (1.09–2.17)§	<0.05**		
		Males	1	37.4%	Larger trochanter, flatter trochanter, a flattening of the transition between femoral head and neck	1.66 (1.11–2.48)	<0.05**		
			2	16.0%	Flattening of the femoral head, somewhat suggestive of cam-type change of femoroacetabular impingement	1.49 (1.01–2.19)	<0.05**		
		With baseline symptoms	6	3.4%	Subtle differences in the size of the greater trochanter, the length of the femoral neck, and the transition between the two†‡	2.11 (1.28–3.50)§	<0.05**		
			14	0.6%	*Not described, not shown in figures*	1.80 (1.06–3.07)§	<0.05**		
		Without baseline symptoms	6	3.4%	Subtle differences in the size of the greater trochanter, the length of the femoral neck, and the transition between the two†‡	1.94 (1.20–3.11)§	<0.05**		
			11	1.1%	Alterations in the transition between greater trochanter and femoral neck	1.52 (1.05–2.17)§	<0.05**		

* These shapes are positively associated with the outcome, unless stated otherwise. For a visual impression of what these shape modes look like, we refer to the original articles. Effect sizes are shown per 1 SD increase in shape mode value. An effect size ratio between 0 and 1 indicates that the negative SDs are associated with the outcome, and ratios above 1 indicate that positive SDs are associated with the outcome. Descriptions in regular typeface are taken literally from the original papers, while descriptions in italics are interpreted from the figures of the original papers;

** Exact *P*-values were not given, but were under the alpha level of0.05;

a This study described a combined outcome definition (THR or KL ≥ 2) in their methods, but only presented KL ≥ 2 cases in their results;

b This study used a combined outcome definition (THR or Croft ≥2);

† This study did not describe what the actual differences between positive and negative SDs were;

‡ In the group *with* baseline symptoms a *decrease* in mode 6 score was associated with the outcome, while in the group *without* baseline symptoms an *increase* in mode 6 score was associated with the outcome;

§ OR for symptomatic radiographic hip osteoarthritis; CI: confidence interval; OR: odds ratio; KL: Kellgren–Lawrence grade; BMI: body mass index; BMD: bone mineral density; SD: standard deviation.
