# Supplementary material for: Statistical shape modeling of the hip and the association with hip osteoarthritis: a systematic review
Source: Osteoarthritis Cartilage. Author manuscript; Available in PMC 2025 Jul 28. (PMC12303122; doi:10.1016/j.joca.2020.12.003)
Supplement: 1 [file NIHMS2026973-supplement-1.pdf]

Supplement 1: Search strategy for the systematic review “Statistical Shape Modeling of the Hip and the Association with Hip Osteoarthritis: a Systematic Review”

Search performed: April 25, 2020

Search yield:

Source		Before deduplication	After deduplication
embase.com		2746	2710
Medline ovid		2621	882
Web of Science Core Collection		2120	775
Cochrane CENTRAL		209	71
Google scholar		200	92
**Total**		**7896**	**4618**

Search terms:

embase.com

('hip osteoarthritis'/de OR (osteoarthritis/de AND ('cam deformity'/de OR 'coxa valga'/de OR 'coxa vara'/de OR 'femoroacetabular impingement'/de)) OR (coxarth* OR ((hip OR hips OR cox) NEAR/6 (osteoarthr* OR arthrit* OR arthrosis* OR arthroses* OR oa)) OR ((osteoarthrit* OR osteo-arthrit*) AND (cam OR 'coxa valga' OR 'coxa vara' OR femoroacetabul*))):ab,ti OR ((hip OR hips OR cox) AND (osteoarthr* OR arthrit* OR arthrosis* OR arthroses* OR oa)):ti) AND ('anatomy'/de OR 'morphology'/de OR 'cam deformity'/de OR 'dysplasia'/de OR 'bone dysplasia'/de OR 'hip dysplasia'/de OR 'coxa valga'/de OR 'coxa vara'/de OR 'femoroacetabular impingement'/de OR 'femur malformation'/de OR 'joint malformation'/de OR 'musculoskeletal system malformation'/de OR 'hip malformation'/de OR 'epiphysiolysis'/exp OR 'coxa magna'/de OR 'bone deformation'/exp OR (anatom* OR morpholog* OR dysplas* OR coxa-valga OR coxa-vara OR (varus NEAR/3 valgus NEAR/3 angle*) OR ((varus OR valgus OR cam OR pincer OR femur OR femoral OR hip OR hips OR cox OR coxae OR acetabul* OR femoroacetabul* OR bone* OR osseous) NEAR/6 (deform* OR malform* OR anomal* OR abnormal* OR shape OR geometr* OR retrovers* OR antevers* OR spheric* OR parameter* OR morpholog* OR lesion*)) OR (Acetabul* NEAR/3 Protrus*) OR (Coxa NEAR/3 profund*) OR impingement* OR (acetabul* NEAR/3 (coverage OR overcoverage)) OR pistol-grip OR (slip* NEAR/3 epiphys*) OR epiphysiolysis):ab,ti) AND ('risk factor'/de OR 'risk assessment'/de OR risk/de OR 'attributable risk'/de OR 'pathophysiology'/de OR prediction/de OR etiology/de OR causality/de OR 'disease association'/de OR 'prevalence'/de OR 'epidemiology'/de OR incidence/de OR (risk* OR Physiopatholog* OR pathophysiolog* OR Physio-patholog* OR patho-physiolog* OR prediction OR cause* OR etiolog* OR aetiolog* OR influen* OR Lead*-to OR due-to OR secondary-to OR trigger* OR associat* OR attribut* OR prevalen* OR epidemiolog* OR incidence*):ab,ti) NOT ([animals]/lim NOT [humans]/lim) NOT ([Conference Abstract]/lim AND [1800-2016]/py) AND [English]/lim NOT (juvenile/exp NOT adult/exp)

Medline ovid

(Osteoarthritis, Hip/ OR (osteoarthritis/ AND (coxa valga/ OR coxa vara/ OR femoroacetabular impingement/)) OR (coxarth* OR ((hip OR hips OR cox) ADJ6 (osteoarthr* OR arthrit* OR arthrosis* OR arthroses* OR oa)) OR ((osteoarthrit* OR osteo-arthrit*) AND (cam OR coxa valga OR coxa vara OR femoroacetabul*))).ab,ti. OR ((hip OR hips OR cox) AND (osteoarthr* OR arthrit* OR arthrosis* OR arthroses* OR oa)).ti.) AND ("anatomy and histology".fs. OR Anatomy/ OR Bone Diseases, Developmental/ OR Hip Dislocation/ OR Coxa Valga/ OR Coxa Vara/ OR Femoracetabular Impingement/ OR femur/ab OR Coxa Magna/ OR (anatom* OR morpholog* OR dysplas* OR coxa-valga OR coxa-vara OR (varus ADJ3 valgus ADJ3 angle*) OR ((varus OR valgus OR cam OR pincer OR femur OR femoral OR hip OR hips OR cox OR coxae OR acetabul* OR femoroacetabul* OR bone* OR osseous) ADJ6 (deform* OR malform* OR anomal* OR abnormal* OR shape OR geometr* OR retrovers* OR antevers* OR spheric* OR parameter* OR morpholog* OR lesion*)) OR (Acetabul* ADJ3 Protrus*) OR (Coxa ADJ3 profund*) OR impingement* OR (acetabul* ADJ3 (coverage OR overcoverage)) OR pistol-grip OR (slip* ADJ3 epiphys*) OR epiphysiolysis).ab,ti.) AND (Risk Factors/ OR Risk Assessment/ OR risk/ OR physiopathology.fs. OR etiology.fs. OR Causality/ OR Prevalence/ OR Epidemiology.fs. OR Epidemiology/ OR Incidence/ OR (risk* OR Physiopatholog* OR pathophysiolog* OR Physio-patholog* OR patho-physiolog* OR prediction OR cause* OR etiolog* OR aetiolog* OR influen* OR Lead*-to OR due-to OR secondary-to OR trigger* OR associat* OR attribut* OR prevalen* OR epidemiolog* OR incidence*).ab,ti.) NOT (exp animals/ NOT humans/) NOT (news OR congres* OR abstract* OR book* OR chapter* OR dissertation abstract*).pt. AND english.la. NOT ((exp child/ OR exp infant/ OR adolescent/) NOT (exp adult/))

Web of Science Core Collection

(TS=(coxarth* OR ((hip OR hips OR cox) NEAR/5 (osteoarthr* OR arthrit* OR arthrosis* OR arthroses* OR oa)) OR ((osteoarthrit* OR osteo-arthrit*) AND (cam OR "coxa valga" OR "coxa vara" OR femoroacetabul*))) OR TI=((hip OR hips OR cox) AND (osteoarthr* OR arthrit* OR arthrosis* OR arthroses* OR oa))) AND TS=((anatom* OR morpholog* OR dysplas* OR coxa-valga OR coxa-vara OR (varus NEAR/2 valgus NEAR/2 angle*) OR ((varus OR valgus OR cam OR pincer OR femur OR femoral OR hip OR hips OR cox OR coxae OR acetabul* OR femoroacetabul* OR bone* OR osseous) NEAR/5 (deform* OR malform* OR anomal* OR abnormal* OR shape OR geometr* OR retrovers* OR antevers* OR spheric* OR parameter* OR morpholog* OR lesion*)) OR (Acetabul* NEAR/3 Protrus*) OR (Coxa NEAR/3 profund*) OR impingement* OR (acetabul* NEAR/2 (coverage OR overcoverage)) OR pistol-grip OR (slip* NEAR/2 epiphys*) OR epiphysiolysis)) AND TS=((risk* OR Physiopatholog* OR pathophysiolog* OR Physio-patholog* OR patho-physiolog* OR prediction OR cause* OR etiolog* OR aetiolog* OR influen* OR Lead*-to OR due-to OR secondary-to OR trigger* OR associat* OR attribut* OR prevalen* OR epidemiolog* OR incidence*) NOT ((child* OR infan* OR adolescen* OR pediatr* OR paediatr*) NOT (adult*))) AND DT=(article) AND LA=(english)

Cochrane CENTRAL

((coxarth* OR ((hip OR hips OR cox) NEAR/6 (osteoarthr* OR arthrit* OR arthrosis* OR arthroses* OR oa)) OR ((osteoarthrit* OR osteo next arthrit*) AND (cam OR 'coxa valga' OR 'coxa vara' OR femoroacetabul*))):ab,ti OR ((hip OR hips OR cox) AND (osteoarthr* OR arthrit* OR arthrosis* OR arthroses* OR oa)):ti) AND ((anatom* OR morpholog* OR dysplas* OR coxa next valga OR coxa next vara OR (varus NEAR/3 valgus NEAR/3 angle*) OR ((varus OR valgus OR cam OR pincer OR femur

OR femoral OR hip OR hips OR cox OR coxae OR acetabul* OR femoroacetabul* OR bone* OR osseous) NEAR/6 (deform* OR malform* OR anomal* OR abnormal* OR shape OR geometr* OR retrovers* OR antevers* OR spheric* OR parameter* OR morpholog* OR lesion*)) OR (Acetabul* NEAR/3 Protrus*) OR (Coxa NEAR/3 profund*) OR impingement* OR (acetabul* NEAR/3 (coverage OR overcoverage)) OR pistol next grip OR (slip* NEAR/3 epiphys*) OR epiphysiolysis):ab,ti) AND ((risk* OR Physiopatholog* OR pathophysiolog* OR Physio next patholog* OR patho next physiolog* OR prediction OR cause* OR etiolog* OR aetiolog* OR influen* OR Lead* next to OR due next to OR secondary next to OR trigger* OR associat* OR attribut* OR prevalen* OR epidemiolog* OR incidence*):ab,ti)

Google scholar

coxarthrosis|"hip osteoarthritis|arthritis|arthrosis|osteo" anatomy|morphology|dysplasia|"coxa valga|vara"|"varus|valgus|cam|pincer deformity|malformation|necrosis|anomaly" risk|Physiopathology|pathophysiology|prediction|etiolog|prevalence|epidemiology
